# Scrotal Pain Caused by a Segmental Testicular Infarct

**DOI:** 10.5811/cpcem.2021.10.53840

**Published:** 2022-01-15

**Authors:** Michael Muradian, Stephen Fox, Patrice Barish, Brett Todd

**Affiliations:** * Beaumont Health, Department of Emergency Medicine, Royal Oak, Michigan; † Oakland University William Beaumont School of Medicine, Department of Emergency Medicine, Auburn Hills, Michigan

**Keywords:** ultrasound, scrotal pain

## Abstract

**Case Presentation:**

A 44-year-old Black male presented to the emergency department with left scrotal pain. His initial workup did not identify an etiology of his symptoms; however, he returned the following day with worsening pain and a radiology-performed ultrasound then revealed a segmental testicular infarct.

**Discussion:**

Segmental testicular infarcts are a rare, often idiopathic, source of scrotal pain. Diagnosis is made by ultrasound, and repeat imaging may be required if not apparent on initial evaluation. Management is typically conservative although some require surgical intervention.

## CASE PRESENTATION

A 44-year-old Black male with history of diverticulitis and anabolic steroid use presented to the emergency department with the complaint of sudden onset right groin and testicular pain. Vitals on initial presentation were within normal limits. Physical examination showed mild tenderness to the right side of the scrotum without testicular tenderness or a mass. Labs were notable for an elevated white blood cell (WBC) count of 12.8 billion/L (reference range: 3.5–10.1 bil/L), hemoglobin of 18.1 bil/L (14.5–17 bil/L), and urinalysis was unremarkable. A scrotal duplex ultrasound demonstrated scrotal wall thickening along with a small hydrocele but no evidence of torsion. Non-contrast enhanced computed tomography of the abdomen and pelvis showed fat stranding around the prostate. Urology was consulted and recommended symptom control, but there was no acute intervention. The patient was discharged with strict return precautions.

**Image 1:**
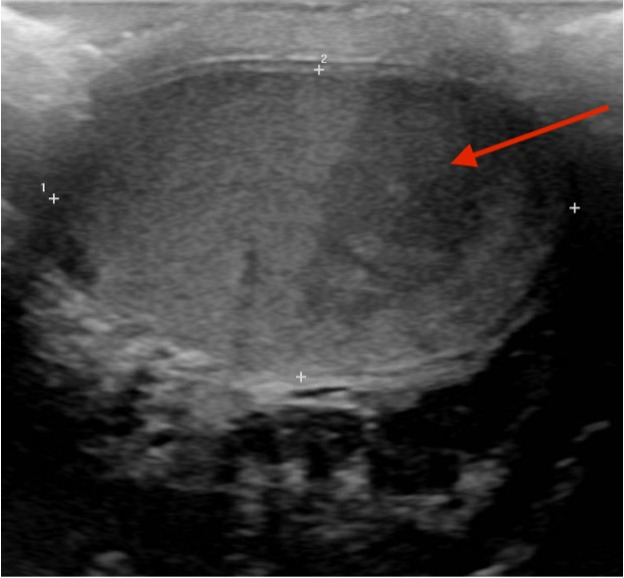
A hypoechoic wedge-shaped area (arrow) seen on ultrasound in the right testicle on sagittal view.

**Image 2:**
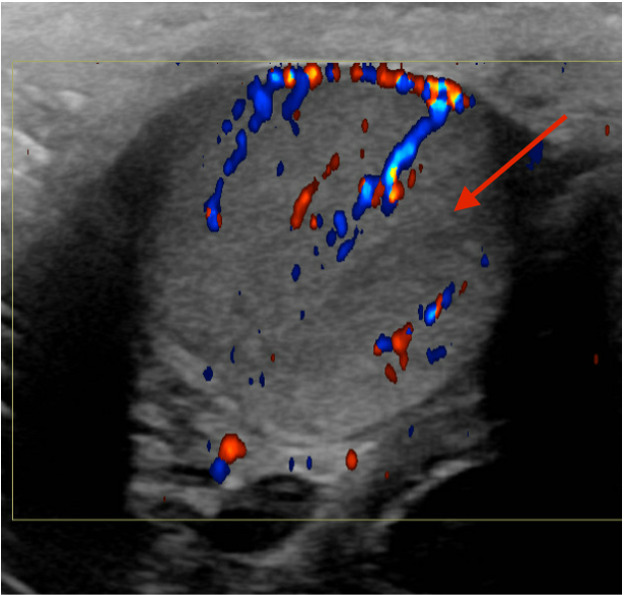
A hypoechoic wedge-shaped area (arrow) showing no flow on ultrasound with Doppler in this transview.

The following day he returned with worsening right scrotal pain. Vitals at that time were notable for tachycardia to 105 beats per minute and a blood pressure of 160/112 millimneters mercury. Exam showed exquisite tenderness to the right lateral testicle. Repeat lab work showed the WBC count had risen to 14.8 bil/L and a hemoglobin of 18.7 bil/L. Although his age and atypical presentation made intermittent torsion appear unlikely, a repeat radiology-performed ultrasound showed a hypoechoic, wedge-shaped abnormality in the right testicle with absent perfusion. The remainder of the testicle had relatively increased vascularity consistent with a segmental testicular infarct (Images 1 and 2). The patient was admitted for further evaluation and was managed conservatively with aspirin. He then had a workup including testing for clotting disorders, other primary or secondary causes of polycythemia, and tumor markers, which was unrevealing. The infarct was attributed to polycythemia caused by long-term anabolic steroid use.

## DISCUSSION

Segmental testicular infarction is a rare but important cause of testicular pain that can mimic other etiologies of scrotal and testicular pain. It is most common in men ages 20–40. While most cases are idiopathic, it is associated with vasculitis, sickle cell disease, trauma, torsion, infection, malignancy, and polycythemia.^1^,^2^ In this case, it is likely the patient’s use of steroids and subsequent polycythemia were the cause of his infarct given the lack of other risk factors. The use of anabolic steroids is known to contribute to polycythemia and increase the risk of thrombosis and ischemic events.^3^

Testicular ultrasound with color Doppler is diagnostic and can differentiate this process from etiologies that present similarly, such as torsion. It typically reveals a hypoechoic area with decreased or absent Doppler flow.^1^ This condition is usually managed conservatively; however, some undergo orchiectomy if their diagnosis is unclear or there is concern for malignancy.^4^ This case highlights steroid use and polycythemia as important risk factors for segmental infarcts, which have not been well reported in the emergency medicine literature. It also illustrates the need for repeat imaging for a patient with persistent or worsening testicular pain as a segmental infarct may not be apparent on initial presentation.

**CPC-EM Capsule**

What do we already know about this clinical entity?

*Segmental testicular infarcts are a rare cause of scrotal pain that are diagnosed by ultrasound with Doppler showing a hypoechoic area with decreased flow*.

What is the major impact of the image(s)?

*These images show the characteristic findings of a segmental testicular infarct of which emergency physicians should be aware*.

How might this improve emergency medicine practice?

*This case highlights a risk factor for testicular segmental infarcts and demonstrates the importance of considering repeat imaging*.
